# Cloning and Functional Characterization of Dog OCT1 and OCT2: Another Step in Exploring Species Differences in Organic Cation Transporters

**DOI:** 10.3390/ijms23095100

**Published:** 2022-05-04

**Authors:** Marleen Julia Meyer, Simon Falk, Sarah Römer, Clarissa Prinzinger, Sabine Tacke, Joachim Geyer, Stefan Simm, Mladen Vassilev Tzvetkov

**Affiliations:** 1 Department of General Pharmacology, Institute of Pharmacology, Center of Drug Absorption and Transport (C_DAT), University Medicine Greifswald, 17487 Greifswald, Germany; famos.link@gmail.com (S.F.); sarah.roemer@med.uni-greifswald.de (S.R.); mladen.tzvetkov@uni-greifswald.de (M.V.T.); 2 Faculty of Veterinary Medicine, Institute of Pharmacology and Toxicology, Justus Liebig University Giessen, 35390 Giessen, Germany; clarissa.prinzinger@gmx.de (C.P.); joachim.m.geyer@vetmed.uni-giessen.de (J.G.); 3 Clinic for Small Animals, Faculty of Veterinary Medicine, Justus Liebig University Giessen, 35390 Giessen, Germany; sabine.p.tacke@vetmed.uni-giessen.de; 4 Institute of Bioinformatics, University Medicine Greifswald, 17487 Greifswald, Germany; stefan.simm@uni-greifswald.de

**Keywords:** organic cation transporter, SLC22A1, SLC22A2, species differences, ortholog comparison, gene structure, metformin, trospium, fenoterol, ipratropium, butylscopolamine

## Abstract

OCT1 and OCT2 are polyspecific membrane transporters that are involved in hepatic and renal drug clearance in humans and mice. In this study, we cloned dog OCT1 and OCT2 and compared their function to the human and mouse orthologs. We used liver and kidney RNA to clone dog OCT1 and OCT2. The cloned and the publicly available RNA-Seq sequences differed from the annotated exon-intron structure of OCT1 in the dog genome CanFam3.1. An additional exon between exons 2 and 3 was identified and confirmed by sequencing in six additional dog breeds. Next, dog OCT1 and OCT2 were stably overexpressed in HEK293 cells and the transport kinetics of five drugs were analyzed. We observed strong differences in the transport kinetics between dog and human orthologs. Dog OCT1 transported fenoterol with 12.9-fold higher capacity but 14.3-fold lower affinity (higher K_M_) than human OCT1. Human OCT1 transported ipratropium with 5.2-fold higher capacity but 8.4-fold lower affinity than dog OCT1. Compared to human OCT2, dog OCT2 showed 10-fold lower transport of fenoterol and butylscopolamine. In conclusion, the functional characterization of dog OCT1 and OCT2 reported here may have implications when using dogs as pre-clinical models as well as for drug therapy in dogs.

## 1. Introduction

Organic cation transporters OCT1 and OCT2 (gene names *SLC22A1* and *SLC22A2*, respectively) are polyspecific membrane transporters with partially overlapping substrate spectra but different patterns of organ expression. OCT1 is predominantly expressed in human hepatocytes where it mediates the first step in hepatic metabolism or excretion [1,2]. OCT2 is primarily expressed in human renal proximal tubules where it is involved in the tubular secretion of organic cationic or weakly basic substances [2]. However, species-specific differences in OCT1 organ expression have been reported. In contrast to human OCT1, rodent OCT1 is expressed both in the liver and in the kidney [3,4,5].

OCT1 and OCT2 substrates are clinically relevant drugs like metformin, fenoterol, sumatriptan, tramadol, ipratropium, and ranitidine [6,7,8,9,10,11,12,13,14,15]. The European Medicines Agency (EMA) and the U.S. Food and Drug Administration (FDA) recommend as a part of pre-clinical drug development to evaluate drug-drug interactions involving OCT2 (both EMA and FDA) and OCT1 (EMA) [16,17]. In 2018, the International Transporter Consortium (ITC) also acknowledged the clinical relevance of OCT1 and recommended its evaluation during drug development [18,19].

For efficacy, toxicity, and safety assessment, the FDA and EMA recommend testing new molecular entities in two independent animal models, usually one rodent and one non-rodent model [20,21]. Despite efforts to reduce animal testing, next to mice and rats, the dog remains a highly relevant animal model. Dogs share similarities with humans in cardiovascular, urogenital, nervous, and musculoskeletal systems and have a natural tendency to develop cancers that share many characteristics with human malignancies [22]. Furthermore, the dog is a popular pet and thereby relevant as a patient in veterinary healthcare. Up to 90 million domestic dogs are estimated to live in both Europe and the U.S., with numbers steadily rising [23,24]. Therefore, a better understanding of the role of dog OCTs in the pharmacokinetics of veterinary medications could improve drug safety for these animals.

Despite the role of organic cation transporters in drug transport and the importance of the dog as a pre-clinical model, there is very little data for dog OCTs in the literature. Drug-metabolizing enzymes have been studied and some drug transporters, such as OATP1B4, have been described in dogs [25,26], but there is only very limited knowledge about dog solute carrier (SLC) transporters. An initial characterization of the expression, regulation, and transport activity of dog OCT2 in Madin–Darby canine kidney (MDCK) model cells was published 20 years ago [27]. However, these analyses focused on endogenously expressed dog OCT2 and on the interaction with model OCT substrates and inhibitors. Meanwhile, the first dog genome was sequenced and annotated (CanFam3.1, https://www.ncbi.nlm.nih.gov/assembly/GCF_000002285.3/, accessed on 22 November 2019), but there is little to no data about the expression and functional characterization of dog OCTs. Furthermore, to the best of our knowledge, no functional data for dog OCT1 have been reported so far.

Another aspect of potential interest for dog OCTs is that despite extensive research on OCT1 and OCT2 during the past 25 years, the exact amino acids involved in substrate binding and/or translocation, and the mechanism conferring their polyspecificity, are unclear. One strategy for revealing the mechanisms of transport and polyspecificity is the analysis of the functional differences between OCT1 orthologs in mammals [28]. Using this strategy for human and mouse OCT1, we identified amino acid differences in transmembrane helix 2 (TMH2) and TMH3 that confer differences in the affinity for metformin [29], and the amino acid differences at codons 32 and 36 confer differences in the affinity for trospium and fenoterol, respectively [30]. The same approach may be applied to the dog orthologs.

The aim of this study was to clone and functionally characterize dog OCT1 and OCT2, and to analyze their expression in the dog liver and kidney. This will help translate data involving pre-clinical research on OCT1 and OCT2 in dog animal models to humans, and improve drug safety for dogs as patients. Moreover, systematic comparisons of dog OCT1 and OCT2 with their human orthologs may provide new insights into the transport mechanisms for this group of transporters.

## 2. Results

### 2.1. Gene Structure of Dog OCT1 and OCT2

**Figure 1:**
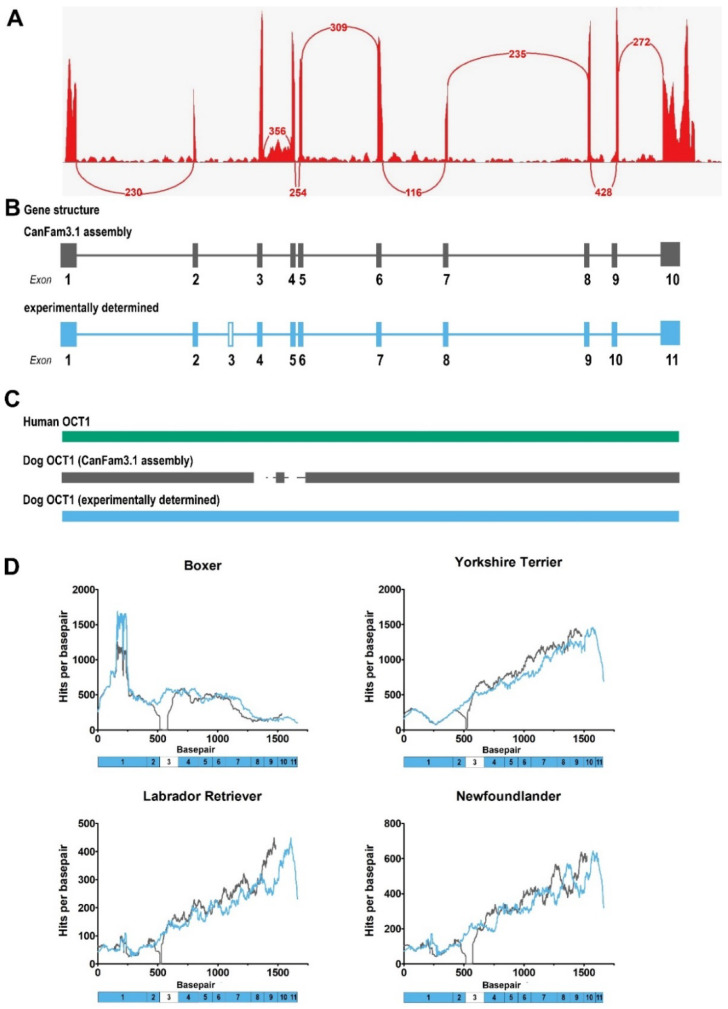
Gene structure of dog OCT1. (**A**) Sashimi plot showing OCT1 (SLC22A1) RNA-Seq reads from Beagle dog liver mapped onto chromosome 1 of the CanFam3.1 dog genome assembly. Exon junctions based on spliced reads are connected by lines, with the number indicating the number of splice junctions mapped. RNA-Seq dataset PRJEB33381 from ENA at EMBL-EBI was used. (**B**) Exon-intron structure of dog OCT1 according to the CanFam3.1 genome assembly (top) and our experimental determination (bottom). The experimentally determined structure contains an additional exon between exons 2 and 3 of the CanFam3.1 genome assembly. (**C**) Schematic protein sequence alignment of human OCT1 (green) and dog OCT1 according to CanFam3.1 (grey) and our experimental results (blue). (**D**) Comparison of RNA-Seq coverage mapped onto dog OCT1 cDNA sequences according to CanFam3.1 (grey) and our experimental determination (blue). The x-axis numbering represents the cDNA sequence based on the experimentally determined dog OCT1 sequence and the exon structure shown below. RNA-Seq datasets PRJNA396033 (Newfoundlander, Labrador Retriever, and Yorkshire Terrier) and PRJNA601830 (Boxer) from NCBI GEO were used.

We noticed that the annotated genomic sequence of dog OCT1 (*SLC22A1*) in the currently available *Canis lupus familiaris* genome assembly CanFam3.1 (Breed: Boxer, NCBI) had one exon less than OCT1 orthologs in other mammals. To validate this annotation, we bioinformatically analyzed the gene structure of dog OCT1 using RNA-Seq datasets from dog liver that were already available in the public databases NCBI GEO [31] and EBI ArrayExpress [32]. The available RNA-Seq datasets were splice-aware mapped onto the first chromosome of the CanFam3.1 dog genome. The resulting sashimi plot by IGV [33] showed coverage and presentation of introns verified by splice junctions except between exons 2 and 3 (Figure 1A,B). Interestingly, a high coverage of reads could be observed in the annotated intron 3. Similarly, a high coverage of reads and a missing splice connection between exons 2 and 3 were observed when analyzing another five dog breeds (Figure S1), suggesting a misannotation of the OCT1 gene structure or an incomplete dog genome.

In contrast, when we mapped RNA-Seq datasets from dog kidney for OCT2 using the same reference genome (CanFam3.1), no differences between the annotated dog OCT2 gene structure (CanFam3.1) and the RNA-Seq datasets for kidney could be observed (Figure S2). This suggests first, that the annotation of dog OCT2 in the genome is correct, and second, verified the methodological bioinformatics approach used.

### 2.2. Cloning of Dog OCT1 and OCT2 mRNA Transcripts from Dog Liver and Kidney

To verify the observed exon-intron structure of dog OCT1, we cloned OCT1 from dog liver and OCT2 from dog kidney cDNA and sequenced the complete open reading frames (ORFs). Compared with the published annotated ORF sequence from the CanFam3.1 genome assembly, an additional 87 bp in the exon 3 region were observed, which code for an additional 21 amino acids, resulting in a total of 554 amino acids, which is the same length as the known human OCT1 protein (Figure 1B,C).

To bioinformatically validate the experimentally determined dog OCT1 mRNA sequence, the mapping of liver RNA-Seq datasets from different dog breeds were compared between the annotated (CanFam3.1) and the experimentally determined mRNA sequence. Four different datasets were used: Boxer, Yorkshire Terrier, Labrador Retriever, and Newfoundlander. As expected, when mapping onto the CanFam3.1 reference, there was a short stretch without coverage after approximately 500 bp in all four dog breeds analyzed, and the coverage profile was replaced after this position compared to the reads mapped onto the bioinformatically validated sequence (Figure 1D). By this approach, we could confirm that the correct dog OCT1 sequence differs from the annotated dog genome assembly in the NCBI database (CanFam3.1).

**Figure 2:**
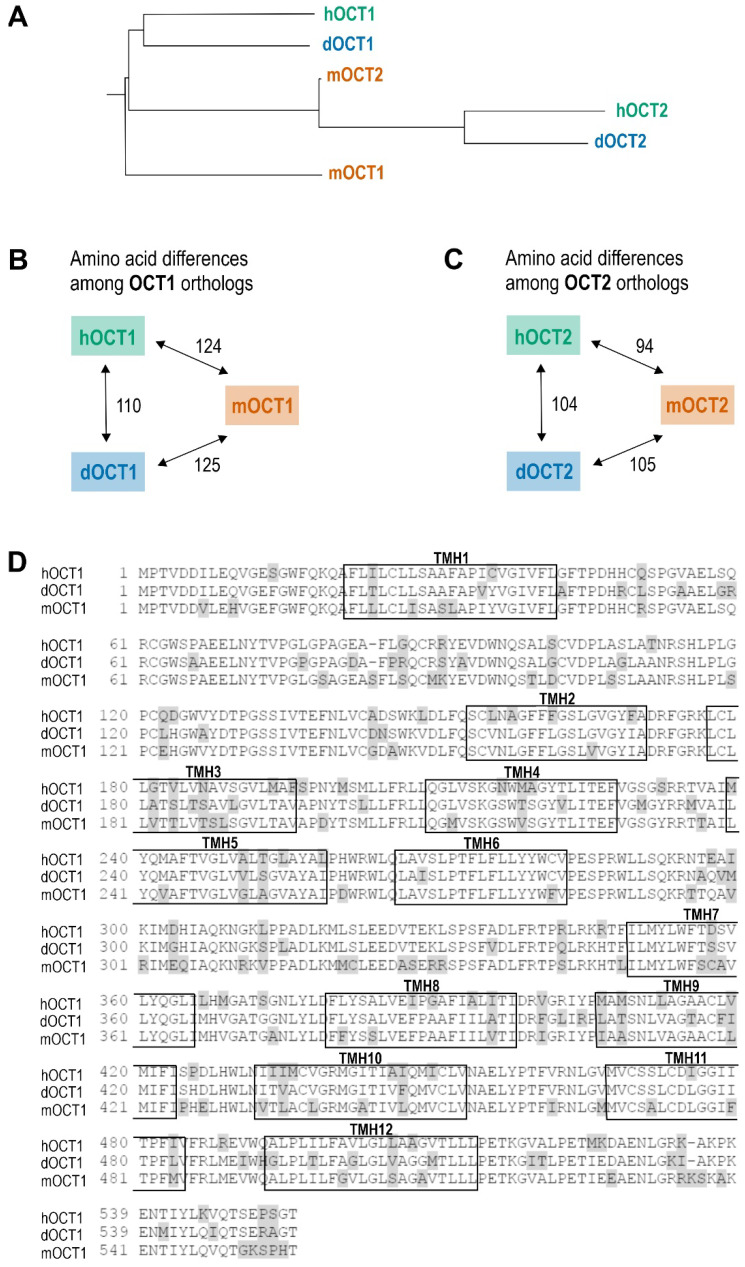
Protein sequence comparison of OCT1 and OCT2 orthologs of human, mouse, and dog. (**A**) Phylogenetic tree showing the similarities of human, dog, and mouse OCT1 and OCT2 protein sequences. Branch lengths not to scale. (**B**,**C**) Number of amino acid differences between dog, human, and mouse OCT1 (**B**) and OCT2 (**C**). (**D**) Alignment of human, dog, and mouse OCT1 protein Scheme 1. are shown in boxes. Sequences were compared using clone manager suite v.9.0 and the BLOSUM62 algorithm.

The experimentally determined dog OCT1 sequence shares 80% amino acid identity with human OCT1, which is greater than the mouse ortholog (which has 77% identity to human) and leaves 110 non-synonymous amino acids between dog and human OCT1 (compared to 124 between mouse and human; Figure 2).

In contrast to OCT1, the cloned dog OCT2 sequence was identical to the annotated OCT2 of CanFam3.1 as well as to the contigs from RNA-Seq datasets and encodes a protein of 533 amino acids. Dog OCT2 shares 81% amino acid identity with human OCT2 and 83% amino acid identity with mouse OCT2 (Figure 2). 

### 2.3. Organ Expression of Dog OCT1 and OCT2

**Figure 3:**
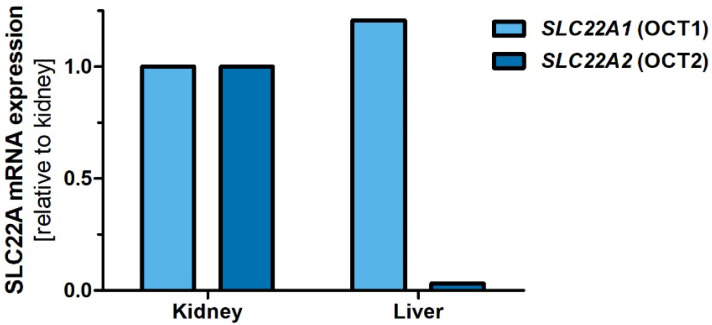
Organ expression of OCT1 and OCT2 mRNAs in dog liver and kidney. Expression of OCT1 (light blue) and OCT2 (dark blue) was measured in dog liver and kidney cDNAs using RT-qPCR. Expression was normalized to the expression of TBP and related to the expression in the kidney.

We analyzed the expression of dog OCT1 (*SLC22A1*) and OCT2 (*SLC22A2*) in liver and kidney samples using RT-qPCR. Dog OCT1 was highly expressed in both the liver and the kidney, and therewith, had a similar organ expression pattern as the rodent, but not the human, OCT1 ortholog. In contrast, dog OCT2 was highly expressed in the kidney, but not in the liver, and therewith, had similar organ expression as the human and rodent orthologs (Figure 3).

### 2.4. Comparative Characterization of Transport Kinetics between Human and Dog OCT1

**Figure 4:**
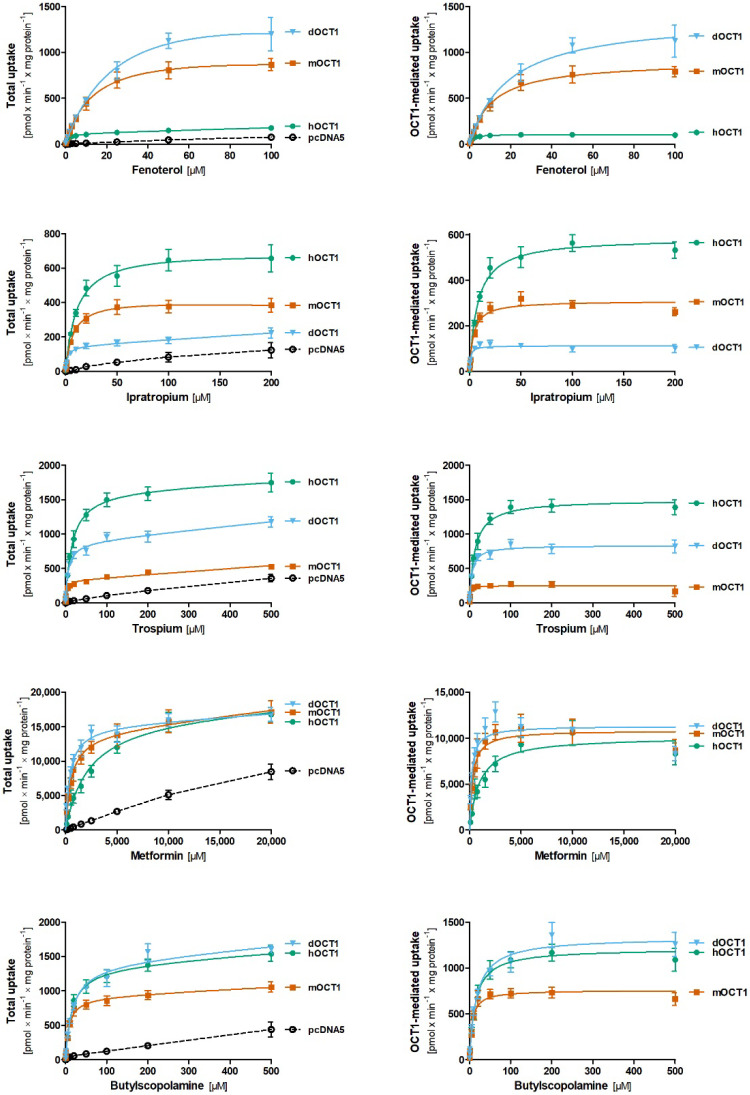
Comparative characterization of substrate uptake between dog, human, and mouse OCT1 orthologs. HEK293 cells stably transfected to overexpress dog (blue), human (green), and mouse (red) OCT1 were incubated for 2 min with increasing concentrations of fenoterol, ipratropium, trospium, metformin, and butylscopolamine. The total uptake is shown for the overexpressing and control cells (left hand side). OCT1-mediated uptake (right hand side) was calculated by subtracting the uptake of control cells (pcDNA5) from the uptake of OCT1-overexpressing cells. The means and standard errors of the means are shown for at least three independent experiments. An Eadie–Hofstee transformation of the data is shown in Figure S3.

**Figure 5:**
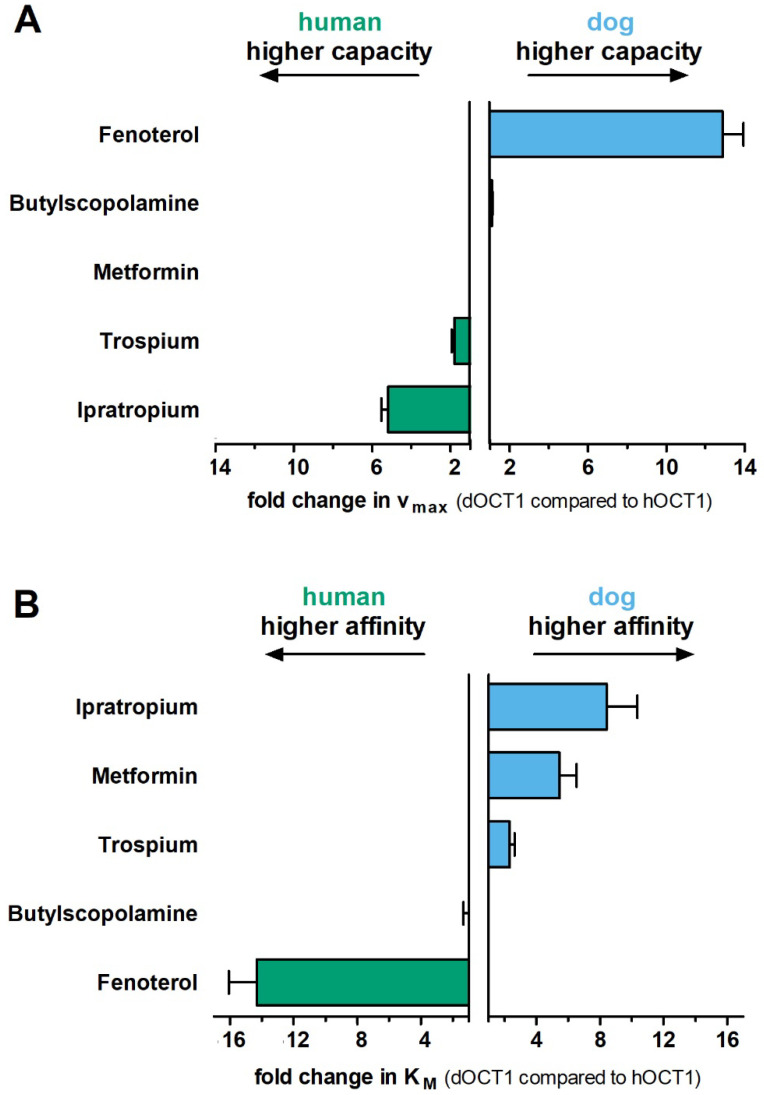
Comparison of the transport capacity (v_max,_ (**A**)) and affinity (K_M_, (**B**)) between dog and human OCT1 orthologs. The fold change in v_max_ and K_M_ between dog (blue) and human (green) OCT1 are shown for the substrates from Figure 4. The means and standard errors of the means are shown for at least three independent experiments.

We functionally characterized dog OCT1 by using stably overexpressing HEK293 cells (for cell generation and validation, see Supplementary Methods Figure S7). We compared the uptake of five clinically relevant OCT1 substrates (fenoterol, ipratropium, trospium, metformin, and butylscopolamine) between dog, mouse, and human OCT1 orthologs. Concentration-dependent uptake measurements showed substrate-specific differences in the transport kinetics between the orthologs that affected both the maximal transport capacity (v_max_) and affinity (K_M_; Figure 4, Figure 5 and Figure S3, Table 1).

Comparing dog and human OCT1, fenoterol and ipratropium were the most extreme examples. Fenoterol was transported by dog OCT1 with 12.9-fold higher capacity but 14.3-fold lower affinity (higher K_M_) than human OCT1 (Figure 4 and Figure 5, Table 1). In contrast, ipratropium was transported by human OCT1 with 5.2-fold higher capacity and 8.4-fold lower affinity than dog OCT1. Similarly, although less extreme than ipratropium, trospium was transported by dog OCT1 with 1.8-fold higher capacity and 2.3-fold lower affinity than human OCT1. Interestingly, metformin was transported with similar capacity but with 5.5-fold higher affinity by dog OCT1 than human OCT1. Butylscopolamine was the only compound that showed no significant differences in uptake kinetics between dog and human OCT1.

Comparing dog and mouse OCT1, trospium and ipratropium were the most extreme examples. Trospium was transported with 3.4-fold higher capacity and 4.4-fold lower affinity by dog than mouse OCT1 (Figure 4 and Figure S4, Table 1). Similarly, butylscopolamine was transported with 1.8-fold higher capacity and 2.9-fold lower affinity by dog than mouse OCT1. In contrast, ipratropium was transported with 2.7-fold higher capacity and 4.1-fold lower affinity by mouse than dog OCT1. Transport of fenoterol and especially metformin did not differ substantially between these two orthologs.

To exclude effects of differences in OCT1 protein expression between the cell lines on the determination of v_max_ values, we normalized the v_max_ values to the v_max_ of metformin, the best studied substrate of OCT1 and OCT2. This normalization did not change the differences in transport capacity observed between the orthologs (Figure S4).

### 2.5. Comparative Characterization of Transport Kinetics between Human and Dog OCT2

**Figure 6:**
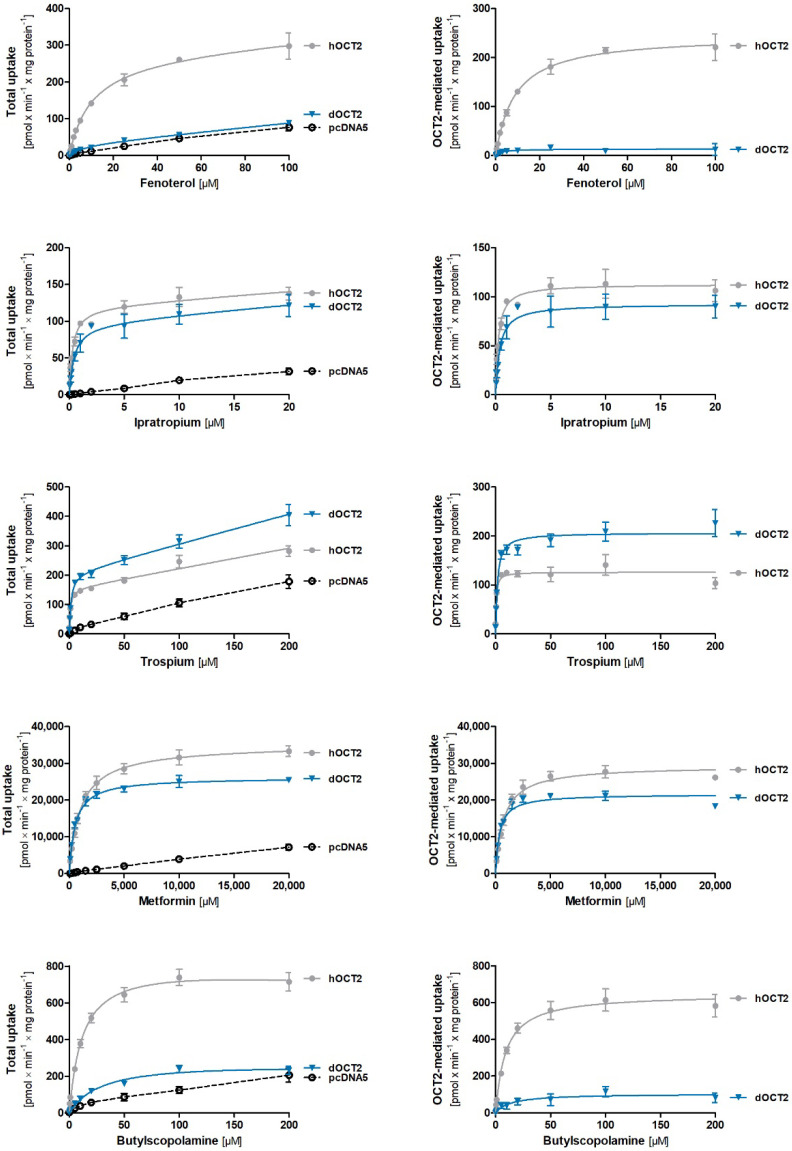
Comparative characterization of substrate uptake between dog and human OCT2 orthologs. HEK293 cells stably transfected to overexpress dog (blue) and human (grey) OCT2 were incubated for 2 min with increasing concentrations of fenoterol, ipratropium, trospium, metformin, and butylscopolamine. The total uptake is shown for the overexpressing and control cells (left hand side). OCT2-mediated uptake (right hand side) was calculated by subtracting the uptake of control cells (pcDNA5) from the uptake of OCT2-overexpressing cells. The means and standard errors of the means are shown for at least three independent experiments. An Eadie–Hofstee transformation of the data is shown in Figure S6.

We functionally characterized dog OCT2 by using stably overexpressing HEK293 cells (for cell generation and validation, see Supplementary Methods Figure S7). We compared the uptake of the same five drugs (fenoterol, ipratropium, trospium, metformin, and butylscopolamine), this time between dog and human OCT2. Concentration-dependent uptake measurements showed substrate-specific differences in the transport kinetics between dog and human OCT2 orthologs (Figure 6 and Figure S6, Table 2) that affected the maximal transport capacity and affinity.

The most prominent differences were observed for fenoterol and butylscopolamine. In contrast to human OCT2, dog OCT2 showed only a slight increase in fenoterol and butylscopolamine uptake compared to control cells, especially at low concentrations, and the data could not be fitted to the Michaelis–Menten equation (Figure 6). Trospium was transported by dog OCT2 with 5.7-fold lower affinity but similar capacity than human OCT2. Metformin and ipratropium were transported with similar capacity and affinity by dog and human OCT2.

### 2.6. Variability of OCT1 Sequence and Expression among Different Dog Breeds

In addition to the newly identified 87 bp in the exon 3 region, the experimentally determined dog OCT1 sequence from a Beagle had one amino acid substitution compared to the published sequence for a Boxer from the CanFam3.1 assembly. This suggested genetic variability among or within different dog breeds.

**Figure 7:**
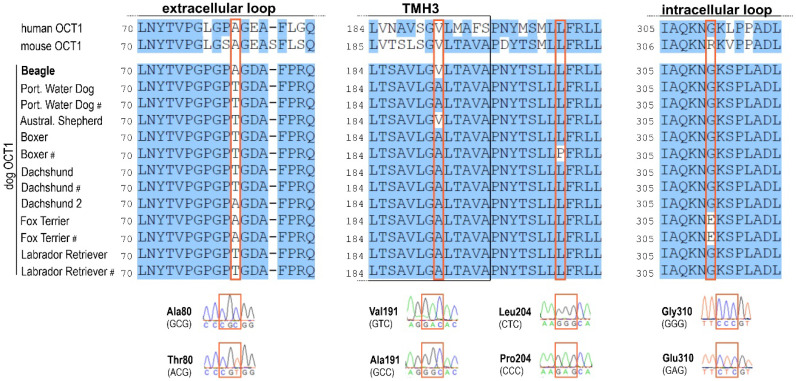
Comparison of OCT1 genetic variability among dogs from different breeds and comparison with human and mouse OCT1s. Dog liver cDNAs from eight individual dogs with seven different breeds were used for re-sequencing of the whole OCT1 open reading frame. Genetic polymorphisms corresponding to amino acid substitutions were found in the large extracellular loop, transmembrane helix 3 (TMH3), and the large intracellular loop. Affected codons are marked in boxes. Exemplary electropherograms depicting the sequence variation are also shown. The experimentally validated reference sequence for the Beagle is highlighted in bold. In five cases, two clones from one breed were sequenced and the second clone is denoted by “#”. For Dachshund, two individual dogs were analyzed.

To further analyze genetic variability in OCT1 among dogs, dog OCT1 cDNA from eight dog liver samples originating from seven different breeds was cloned and 13 clones (comprising three clones from two Dachshund donors, one clone from a Beagle and an Australian Shepherd, and two clones from one donor for the rest of the breeds) were completely re-sequenced. The dog OCT1 sequences revealed the same length of 554 amino acids and very high amino acid identity among the different dogs analyzed (99%; Figure 7). There were four codons where individual dogs carried an amino acid substitution compared to the validated Beagle OCT1 sequence: codons 80 (extracellular loop), 191 (TMH3), 204 (between TMH3 and 4), and 310 (intracellular loop). The Beagle sequence shared Ala80 with a Fox Terrier and human and mouse OCT1, whereas the Labrador Retriever, Portuguese Water Dog, Australian Shepherd, Boxer, and Dachshund possessed Thr80. Val191 in Beagle OCT1 was shared with an Australian Shepherd dog, as well as with human and mouse OCT1, while all the other dogs showed Ala191 at this position. At codons 204 and 310, the Boxer and the Fox Terrier possessed Leu204Pro and Gly310Glu substitutions, respectively.

**Figure 8:**
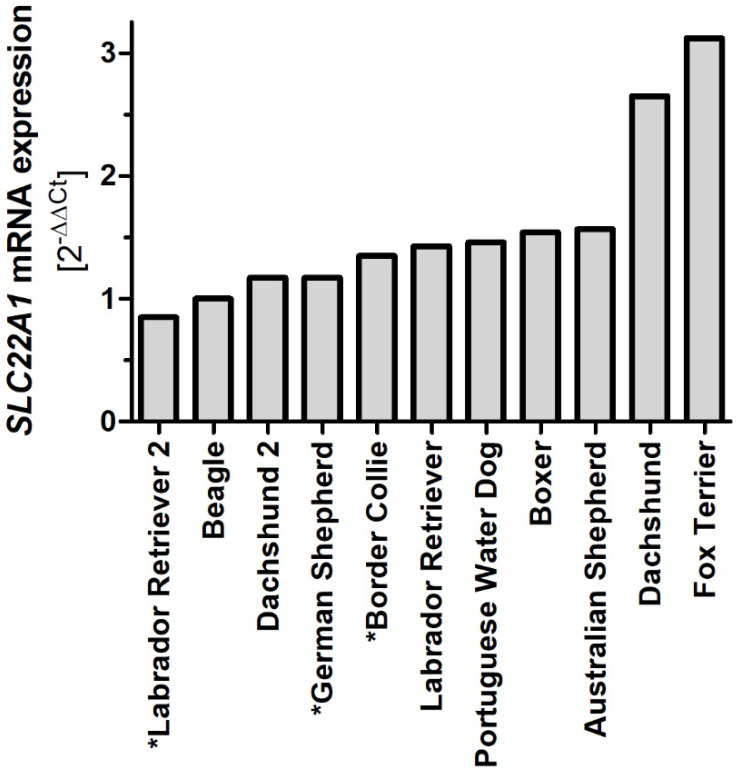
Comparison of OCT1 mRNA expression in liver samples from different dogs. Expression of OCT1 (*SLC22A1*) was measured in cDNA from eleven liver samples originating from eight different breeds using RT-qPCR. Expression was normalized to the expression of TBP and Beagle was used as reference. * Denotes samples that were not cloned and sequenced.

Furthermore, we analyzed the variability in dog OCT1 mRNA expression in liver samples from eleven individual dogs with nine different breeds. Most dogs showed similar OCT1 expression levels to the Beagle, ranging from 0.85 for Labrador Retriever 1 to 1.57 for the Australian Shepherd (Figure 8). However, Dachshund 1 had 2.65- and the Fox Terrier had 3.12-fold higher OCT1 expression than the Beagle.

## 3. Discussion

In this study, we report a new annotation for the dog OCT1 gene and describe functional differences for cloned dog OCT1 and OCT2 after overexpression in HEK293 cells in comparison with their human orthologs.

**Figure 9:**
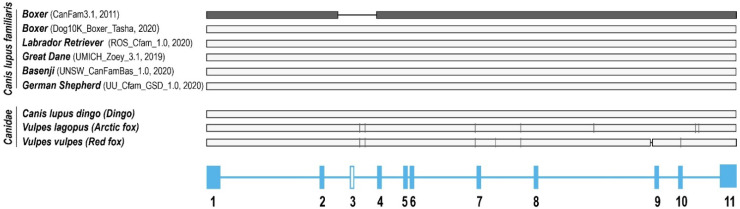
Alignment of dog OCT1 against different canine genomic DNA sequences. The dog OCT1 genomic sequence from the CanFam3.1 assembly (dark grey) has a 2153 bp gap in the exon 3 region compared with more recent dog genome assemblies that are identical to our experimentally determined dog OCT1 sequence. The dog OCT1 exon-intron structure is shown below for orientation.

The cloned cDNA sequence of dog OCT1 differed by 87 bp from the predicted mRNA sequence from the annotation of the primary assembly of dog chromosome 1 in the dog genome CanFam3.1, which was the dog genome available when we started this study. Both bioinformatic analyses and experimentally cloned sequences strongly suggested that the CanFam3.1 genome was incomplete. An additional exon, exon 3, identified experimentally was missing at the genomic DNA level in the CanFam3.1 genome, suggesting that more than 87 bp is missing from the CanFam3.1 assembly. Indeed, new dog genome assemblies, which became available during the preparation of this work (Figure 9), contain an additional 2153 bp sequence. This 2153 bp DNA region harbors the “missing” exon 3 and this sequence is highly similar to the closely related dingo (*Canis lupus dingo*), arctic fox (*Vulpes lagopus*), and red fox (*Vulpes vulpes*; Figure 9). Furthermore, the resulting dog OCT1 protein sequence showed higher homology with the previously known mammalian sequences and the expected secondary structure with 12 transmembrane helices.

One important lesson from this part of our work is that in the era of using de novo synthesis of a whole ORF instead of cDNA cloning, one should be very critical of using bioinformatics data such as genome annotations without validation. A complete de novo OCT1 ORF synthesis based on the CanFam3.1 assembly would have led to analyzing an erroneous protein and incorrect conclusions. On the other hand, we also showed here that using publicly available bioinformatic resources like RNA-Seq databases can prevent such errors, and may support experimental data additionally. Breed-specific genetic variants in dogs have been reported before [34]. In this study, however, Boxer breed-specific differences cannot account for the “missing” sequence since another Boxer genome (Dog10K_Boxer_Tasha; Figure 9) and an RNA-Seq dataset from a Boxer (Figure 1D) both apparently contained the complete sequence. However, inter-individual genetic variability within the Boxer breed cannot be excluded.

In the second part of this study, we functionally characterized cloned dog OCT1 and OCT2. This is relevant, since dogs are both pre-clinical animal models and pets that are treated with drugs as veterinary patients. One limitation of animal models are species-specific differences in organ expression of drug-metabolizing enzymes and transporters. Here, we demonstrated that dog OCT1 is equally strongly expressed in kidney and liver (Figure 3), which is similar to the organ expression of OCT1 in rodents [3,4,5], but substantially different from humans [1,2,8,35]. Differences in organ expression of OCT1 between humans and rodents are well known, but this is the first study reporting such differences between dog and human OCT1. These differences are one aspect that should be considered when translating pharmacokinetics data from dogs to humans.

Species-specific differences in substrate selectivity between OCT orthologs have been suggested before [29,36,37,38], but systematic comparisons are still scarce. To the best of our knowledge, this is the first study reporting functional analyses of dog OCT1. The only study of dog OCTs characterized OCT2 that is endogenously expressed in MDCK model cells [37]. Dresser et al. suggested differences in dog OCT2 transport characteristics compared to other orthologs based on the transport kinetics of the model substrate TEA^+^ compared to previous works. In this study, we confirmed and extended this claim by analyzing a broader spectrum of clinically relevant drugs as OCT2 substrates.

We also report substantial differences in the transport characteristics between dog and human OCT1 and OCT2. For OCT1, the strongest differences in both capacity and affinity were observed in the kinetics of ipratropium and fenoterol transport, and to a lesser extent, for trospium. However, this may not lead to substantial differences in the pharmacokinetics of these drugs between the two species. In spite of a 14.3-fold higher affinity for fenoterol, differences in the transport capacity in the opposite direction (12.9-fold lower) resulted in very similar intrinsic clearances by dog and human OCT1 (80.7 and 88.3 µL × min^−1^ × mg protein^−1^, respectively). Thus, differences in affinity and capacity in the opposite direction may neutralize each other at clinically relevant (low) concentrations. Interestingly, the intrinsic clearance of fenoterol of mouse OCT1 is also very similar to that of dog and human OCT1 (88.5 µL × min^−1^ × mg protein^−1^). As a consequence, effects of OCT1 knockout on the hepatic uptake of fenoterol in mice [38] may closely reflect the effects of OCT1 not just in humans [30] but also in dogs. Compared to human OCT1, dog OCT1 had a 5.5-fold higher affinity but a similar capacity for metformin transport, resulting in a 5.8-fold higher intrinsic clearance (Figure 4, Table 1). Similar differences in intrinsic clearance were reported by us for mouse and human OCT1. Based on the differences measured in vitro, we estimated metformin concentrations in the mouse liver to be up to 11-fold higher than in the human liver [29]. The data reported here suggest that also for dogs, hepatic metformin concentrations may be higher than in humans. This warrants attention when interpreting both mouse and dog data regarding the hepatic mechanism of action for metformin.

Our results may have clinical implications, considering that the dog is a popular pet and potential patient. Better knowledge of dog OCTs may improve drug therapy in dogs. Here, we analyzed as a proof-of-principle some drugs that are known substrates for OCTs in other species [12], but are also commonly used to treat dogs. Butylscopolamine and ipratropium are routinely used as spasmolytic and bronchodilator drugs, respectively, in veterinary medicine. The intrinsic clearance of butylscopolamine and ipratropium by dog OCT1 (122 and 80.5 µL × min^−1^ × mg protein^−1^, respectively) is comparably high compared to the intrinsic clearance of fenoterol by human OCT1 (88.3 µL × min^−1^ × mg protein^−1^; Table 1). In humans, OCT1 was shown to have strong effects on the AUC (area under the plasma concentration time curve) and plasma concentrations of fenoterol [9]. Thus, an important role for OCT1 in the pharmacokinetics of butylscopolamine and ipratropium in dogs could also be suggested.

An additional interesting aspect of the data reported in this study is the ability to draw conclusions about structure-to-function relationships based on substrate-specific differences in the transport kinetics among OCT1 orthologs. In a recent study, we identified amino acid differences at codon 32 (Phe vs. Leu) and codon 36 (Cys vs. Tyr) that confer the differences in the affinities for trospium and fenoterol, respectively, between human and mouse OCT1 [30]. This was supported by docking fenoterol into the available alphafold structural models for human and mouse OCT1. In line with this, the results from this study show that dog OCT1 has a more comparable affinity for trospium and shares Phe32 with human OCT1 (compared with mouse OCT1 that has Leu32). Similarly, dog OCT1 has a more comparable affinity for fenoterol and shares Tyr36 with mouse OCT1 (compared with human OCT1 that has Cys36; Figure 4). In another recent study, we identified differences at codon 155 (corresponding to codon 156 in mouse; Leu vs. Val) together with amino acid(s) located in TMH3 that confer differences in metformin affinity between human and mouse OCT1 [29]. The results from this study show dog and mouse, but not human, OCT1 have very similar affinity for metformin transport (Figure 4), which supports the suggested important role for codon 155/156, since dog and mouse share valine, whereas human has leucine at this position.

There are differences in the affinity for ipratropium between dog and human OCT1 orthologs that cannot be explained by the differences in amino acids at codons 32, 36, and 155/156. This suggests there are still unknown interactions that warrant further and more detailed analyses. Functional differences between OCT2 orthologs are completely unexplored, but are partially very strong, as in the case of fenoterol or butylscopolamine (Figure 6). More detailed analyses using ortholog or paralog chimeric OCT proteins should provide further insights into the transport mechanism and polyspecificity of OCT1 and OCT2.

Additional analyses of the transport characteristics using Eadie–Hofstee transformation of the data (Figures S3 and S6) do not indicate substantial species-specific differences in the mode of transport. For most substrates, a linear profile corresponding to the classical Michaelis–Menten kinetics could be observed. In some cases, such as butylscopolamine uptake by OCT1 and OCT2, a biphasic profile can be suggested. However, one should be careful not to overinterpret this data in cases of limited uptake, such as butylscopolamine by dog OCT2.

Interestingly, the most extreme differences in transport kinetics between dog and mouse OCT1 were observed for ipratropium and trospium, which are structurally very similar. Together with butylscopolamine, the tropane alkaloids trospium and ipratropium share the classical ester of a tropane ring with either one (ipratropium and butylscopolamine) or two phenol rings (trospium) and different N-substitutions. Butylscopolamine and especially trospium are both transported by dog OCT1 with higher capacity and lower affinity than mouse OCT1 (Figure 4 and Figure S4) despite their structural differences in the number of phenol rings. This may suggest that the less bulky N-substitution at the tropane ring present in ipratropium may be favorable for a higher affinity but lower capacity of transport by dog OCT1 than mouse OCT1. This approach comparing the transport kinetics of structurally highly similar compounds (“ligand structure walking”) may be a useful tool for narrowing down and identifying structural moieties within the ligand that determine its interaction with OCTs, as we have previously reported for the inhibition of OCT1 by morphinan opioids [39]. Moreover, combining the comparison of ligands with similar structures with orthologs that have similar protein sequences may reveal regions that confer differences in transport kinetics on both the ligand and the protein side for a better understanding of OCT polyspecificity.

We observed some genetic variability in OCT1 between individual dogs of different dog breeds that may have clinical relevance. Genetic variability in dog CYP enzymes, such as CYP1A2 and CYP2C41, or the efflux transporter MDR1/P-glycoprotein leading to a loss-of function phenotype are well-known for their effect on the efficacy and safety of drug treatment for dogs [34,40,41,42]. The most well-known is the severe sensitivity to ivermectin and other antiparasitic drugs due to a 4-bp deletion in the canine *MDR1* gene [43,44]. It remains to be analyzed whether the genetic variations we observed for dog OCT1 have consequences on OCT1 function and therefore on the pharmacokinetics and efficacy of drugs that are OCT1 substrates. More detailed analyses of genetic variation and its functional consequences may provide a better understanding of the OCT1 transport mechanism.

In conclusion, functional characterization after cloning and overexpressing of dog OCT1 and OCT2 revealed species-specific differences in the transport kinetics of several clinically relevant OCT substrates, which may have implications for the use of the dog as a pre-clinical animal model as well as drug safety for dogs. Moreover, these differences in OCT1 function can be used as a tool to study the species-specific interactions with OCT substrates to better understand OCT transport and the mechanisms underlying OCT polyspecificity. Finally, this work provides an example where critical evaluation and validation of genomic annotations may be warranted before cloning and functional characterization of membrane transporters.

## 4. Materials and Methods

### 4.1. Reagents

Ipratropium bromide and trospium chloride were obtained from Santa Cruz Biotechnology (Heidelberg, Germany). Trospium-d8 was obtained from Toronto Research Chemicals (TRC, Toronto, ON, Canada). Buformin hydrochloride was obtained from Wako Chemicals (Neuss, Germany). Fenoterol hydrochloride, fenoterol-d6, metformin hydrochloride, butylscopolamine bromide, and atropine were obtained from Sigma-Aldrich (Taufkirchen, Germany).

Dulbecco’s Modified Eagle Medium (DMEM), Hank’s Buffered Salt Solution (HBSS), and additives used for cell culturing were obtained from Life Technologies (Darmstadt, Germany). Poly-d-lysine (1-5 kDa), 2-[4-(2-hydroxyethyl)piperazin-1-yl]ethanesulfonic acid (HEPES), bicinchoninic acid, and copper sulfate pentahydrate were obtained from Sigma Aldrich. Twelve-well plates were obtained from Starlab (Hamburg, Germany) and tissue culture flasks from Sarstedt (Nümbrecht, Germany). Acetonitrile and methanol in LC-MS/MS grade were obtained from LGC Standards (Wesel, Germany), formic acid (LC-MS/MS grade) and sodium chloride were obtained from Merck (Darmstadt, Germany). Sodium dodecylsulfate (SDS, ultrapure) was obtained from AppliChem (Darmstadt, Germany).

### 4.2. Cloning Dog OCT1 and OCT2 from Dog Liver and Kidney

Dog OCT1 and dog OCT2 were cloned from commercially available cDNA from dog liver (OCT1) and kidney (OCT2; both AMS Biotechnology, Abingdon, UK) using PCR. Briefly, primers flanking the 5′ and 3′ end of the mRNA (cDNA) were designed to contain 5′ restriction sites for *Hind*III or *EcoR*V for forward and reverse primers, respectively (Table 3), for subsequent cloning. PCR was carried out using the Hot Start KOD Polymerase Kit (Sigma-Aldrich) with initial denaturation at 95 °C for 2 min, followed by 35 cycles at 95° for 30 sec, the individual annealing temperature (see Table 3) for 30 sec, and elongation at 72 °C for 2 min, followed by a final elongation at 72 °C for 10 min. PCR products were extracted from agarose gels, cloned into the pCR2.1-TOPO TA vector using the TOPO TA cloning kit (Life Technologies) according to the manufacturer’s instructions, and the open reading frame was sequenced using capillary sequencing.

For sequencing of dog OCT1 transcripts from a larger group of dogs, surplus material from diagnostic liver biopsies was collected and stored at −80 °C. This procedure was reviewed and registered by the local Animal Welfare Authorities (Regierungspräsidium Giessen; registration No: V 54 19 c 20 15 h 02 kTV 3/2022). These biopsies were used for RNA isolation and cDNA synthesis as described previously [45] and cDNA was subjected to PCR amplification of the whole open reading frame as outlined above. DNA sequencing was performed by Sanger sequencing using the BigDye Terminator v1.1 Cycle Sequencing Kit according to the manufacturer’s instructions and the Genetic Analyzer 3500xL (both Applied Biosystems) and sequence analysis was performed with Sequencing Analysis software version 6.0.

For transfection of dog OCT1 and OCT2 into HEK293 cells, the PCR products were ligated into the pcDNA5/FRT expression vector (Life Technologies) using T4 ligase after double digestion with *Hind*III and *EcoR*V. The expression vectors were dialyzed using 0.025 µm membrane filters (Merck Millipore), were amplified in *E. coli*, and extracted using the Plasmid Plus Midi Kit (QIAGEN, Hilden, Germany) according to the manufacturer’s instructions.

### 4.3. mRNA Expression Analysis of Dog OCT1 and OCT2

Expression of OCT1 and OCT2 mRNA in dog liver and kidney samples or overexpressing cells was quantified using real-time qPCR. Total RNA from tissues was isolated using TRIzol (Sigma-Aldrich) according to the manufacturer’s instructions and as described previously [45]. Total RNA from overexpressing cells was isolated using the RNeasy Plus Mini Kit (QIAGEN) according to the manufacturer’s instructions. Beagle cDNA was purchased commercially (AMS Biotechnology, Abingdon, UK); cDNA from all other dog breeds was synthesized using the SuperScript III First-Strand Synthesis System (Life Technologies) according to the manufacturer’s instructions and as described previously [45]. The cDNA from overexpressing cells was synthesized using the High-Capacity cDNA Reverse Transcription Kit (Applied Biosystems) according to the manufacturer’s instructions. Real-time qPCR was standardized at the mRNA level. Expression of mRNA was analyzed using TaqMan Gene Expression Master Mix and TaqMan assays (Cf02728707_m1 for dog OCT1, Cf02671927_m1 for dog OCT2, Cf02637231_m1 for dog TBP, and M55654.1 for human TBP from HEK293 cells; all Thermo Fisher) in a total volume of 7 µL according to the manufacturer’s instructions. Samples were measured in triplicate with the QuantStudio 12K Flex Real-Time PCR System and analyzed using QuantStudio 12K Flex software v.1.2.2. Expression of OCT1 and OCT2 were normalized to the expression of the housekeeping gene TATA-box binding protein (TBP) and to the expression in Beagle using the 2^−ΔΔCt^ method [46].

### 4.4. Cell Lines and Cell Culturing

HEK293 cells stably overexpressing human OCT1 or OCT2, mouse OCT1, and dog OCT1 or OCT2 were generated by targeted chromosomal integration using the Flp-In™ system (Life Technologies) as described in detail previously [29,47,48]. For generation and characterization, see the Supplementary Methods, Figure S7, and [29,47]. Cells were cultured in Dulbecco’s Modified Eagle’s Medium (DMEM) supplemented with 10% FBS, 100 U/mL penicillin, and 100 µg/mL streptomycin at 37 °C and 5% CO_2_. Cells were passaged twice a week.

### 4.5. Cellular Uptake Experiments

At 48 h prior to the experiment, 6 × 10^5^ cells were seeded per well in 12-well plates pre-coated with poly-d-lysine.

Cellular uptake experiments were performed at 37 °C and pH 7.4 using Hanks’ Buffered Salt Solution (HBSS) supplemented with 10 mM HEPES (referred to as HBSS+ in the following). Cells were washed with 1 mL pre-warmed (37 °C) HBSS+ and uptake was initiated by adding 400 µL of pre-warmed HBSS+ containing the substrate. Uptake was allowed for exactly 2 min and stopped by adding 2 mL ice-cold HBSS+. Cells were washed twice with 2 mL ice-cold HBSS+ then lysed with 80% acetonitrile supplemented with internal standard. The intracellularly accumulated substrate concentrations were measured using LC-MS/MS as described in the following and normalized to the amount of total protein in the samples as measured using the bicinchoninic acid assay [49].

### 4.6. Quantification of Intracellular Substrate Concentration by LC-MS/MS

Intracellular substrate concentrations were quantified using LC-MS/MS. To this end, the cell lysate was centrifuged at 16,000× *g* for 15 min and 350 µL of supernatant was evaporated to dryness under nitrogen flow at 40 °C. The pellet was reconstituted with 200 µL 0.1% formic acid and between 5 and 10 µL was injected into the LC-MS/MS system (Table S2).

An API4000 QTRAP tandem mass spectrometer with ESI interface (AB SCIEX, Darmstadt, Germany) coupled to a Shimadzu Nexera X2 UHPLC system with LC 30AD pumps and SiL 30AC autosampler (Shimadzu, Duisburg, Germany) was used for analysis. Samples were separated on a Brownlee SPP RP-Amide column (4.6 × 100 mm, 2.7 µm, PerkinElmer, Rodgau, Germany) using a mobile phase of 0.1% (*v*/*v*) formic acid and varying concentrations of organic solvent (Table S2).

### 4.7. Mapping of RNA-Seq Datasets on Dog OCT1 and OCT2 Reference

For splice-aware mapping of RNA-Seq datasets we used the tool HISAT2 [50], and for the coverage of reads, the tool NextGenMap [51]. As reference, we used the genome assembly of CanFam3.1 (GCF_000002285.3 from NCBI) [52,53]. RNA-Seq datasets have been downloaded from the NCBI Gene Expression Omnibus (GEO; [31]) and ArrayExpress [32]. The datasets used for this study were deposited in the NCBI GEO under accession numbers PRJNA415552 (Beagle kidney; [54]), PRJNA396033 (Newfoundlander, Labrador, Yorkshire Terrier, Belgian Malanois), and PRJNA601830 (Boxer) or in the European Nucleotide Archive (ENA) at EMBL-EBI under accession number PRJEB33381 (https://www.ebi.ac.uk/ena/browser/view/PRJEB33381, accessed on 1 April 2020). For mapping, we used default parameter sets from the two tools to perform a mapping to the full reference genome as well as the single genes of interest OCT1 (SLC22A1, gene ID 484068) and OCT2 (SLC22A2, gene ID 403655). Afterwards, the BAM and SAM files were sorted by Picard tools (http://broadinstitute.github.io/picard/, accessed on 10 February 2020) and indexed for visualization by integrative genomics viewer (IGV; [33]). IGV was also used to create the sashimi plots to identify splice junctions and compare the exon-intron structures with the help of HISAT2 output. SAMtools [55] was used with default settings for the basepair coverage of different dog breeds for the two OCT1 and OCT2 gene structures.

### 4.8. Data Analyses

Kinetic transport parameters v_max_ and K_M_ were calculated by non-linear regression with the Michaelis–Menten equation using GraphPad Prism version 5.01 (GraphPad Software Inc., LaJolla, CA, USA). Intrinsic clearance CL_int_ was calculated by dividing v_max_ by K_M_ for each experiment. Kinetic parameters were compared between OCT1 orthologs using an ANOVA followed by Tukey’s honestly significant difference post hoc comparisons in SPSS Statistics version 28 (SPSS Inc., IBM, Chicago, IL, USA).

## Figures and Tables

**Table 1:** Kinetic parameters of OCT1-mediated substrate uptake by dog, human, and mouse OCT1.

	v_max_ [pmol × min^−1^ × mg Protein^−1^] ± SEM			K_M_ [µM] ± SEM			CL_int_ [µL × min^−1^ × mg Protein^−1^] ± SEM		
Substrate	dOCT1	hOCT1	mOCT1	dOCT1	hOCT1	mOCT1	dOCT1	hOCT1	mOCT1
Fenoterol	1346 ± 134	106 ± 11.8 ***	912 ± 78.3 *	16.8 ± 1.09	1.19 ± 0.09 ***	10.7 ± 1.09 *	80.7 ± 7.76	88.3 ± 3.85	88.5 ± 15.5
Ipratropium	113 ± 1.43	586 ± 42.7 ***	309 ± 18.8 *	1.02 ± 0.20	7.79 ± 0.48 ***	3.77 ± 0.31 **	122 ± 28.1	75.7 ± 6.33	83.6 ± 10.4
Trospium	835 ± 64.9	1503 ± 103 ***	255 ± 33.2 ***	5.72 ± 0.30	13.3 ± 1.58 **	1.92 ± 0.85	146 ± 10.3	117 ± 12.7	180 ± 37.5
Metformin	11,373 ± 714	10,465 ± 1306	10,891 ± 1249	204 ± 41.8	1090 ± 297	298 ± 46.9 *	63.1 ± 12.0	11.0 ± 2.05 **	38.7 ± 6.23
Butylscopolamine	1383 ± 112	1249 ± 117	778 ± 59.2 *	18.7 ± 3.32	15.0 ± 1.67	6.60 ± 1.13 *	80.5 ± 12.3	84.4 ± 6.76	131 ± 25.6

* *p* < 0.05, ** *p* < 0.005, *** *p* < 0.001 compared to dOCT1 in a Tukey’s post hoc following ANOVA.

**Table 2:** Kinetic parameter of OCT2-mediated substrate uptake by dog and human OCT2.

	v_max_ [pmol × min^−1^ × mg Protein^−1^] ± SEM		K_M_ [µM] ± SEM		CL_int_ [µL × min^−1^ × mg Protein^−1^] ± SEM	
Substrate	dOCT2	hOCT2	dOCT2	hOCT2	dOCT2	hOCT2
Fenoterol	n.d.	248 ± 21.7	n.d.	8.81 ± 0.55	n.d.	28.0 ± 0.95
Ipratropium	93.2 ± 12.6	114 ± 10.5	0.35 ± 0.02	0.24 ± 0.04 *	265 ± 35.3	477 ± 31.9 *
Trospium	224 ± 14.6	119 ± 13.1 **	2.18 ± 0.27	0.49 ± 0.15 **	106 ± 8.78	278 ± 40.5 *
Metformin	21,578 ± 606	29,650 ± 1457 *	365 ± 11.5	766 ± 95.4 *	59.2 ± 2.76	39.9 ± 5.27 *
Butylscopolamine	n.d.	616 ± 52.0	n.d.	7.62 ± 0.85	n.d.	81.6 ± 2.16

n.d., kinetic parameter could not be determined; * *p* < 0.05, ** *p* < 0.005 compared to dOCT2 in a Tukey’s post hoc following ANOVA.

**Table 3:** Sequences and annealing temperatures of primers used for cloning dog OCT1 and OCT2.

Primer	Sequence (5′-3′)	Annealing Temperature [°C]
dOCT1_HindIII_for dOCT1_EcoRV_rev	GTGATGAAGCTTCTGGCTCCGTTATGCCCACCG	70
	CCGAGCGATATCTCTCTCTCAGGTGCCGGCACG	
dOCT2_for dOCT2_rev	AGCATCGGAAGCTTTCCTGCCTCCGGAGATAATGCCAACT	50
	GTATGGAGGATATCAGCTCCCTACCTCTGCATGTTT	

## Data Availability

Not applicable.

## Supplementary Materials

The following supporting information can be downloaded at: https://www.mdpi.com/article/10.3390/ijms23095100/s1.

## References

[1] Zhang L., Dresser M.J., Gray A.T., Yost S.C., Terashita S., Giacomini K.M. (1997). Cloning and Functional Expression of a Human Liver Organic Cation Transporter. Mol. Pharmacol..

[2] Gorboulev V., Ulzheimer J.C., Akhoundova A., Ulzheimer-Teuber I., Karbach U., Quester S., Baumann C., Lang F., Busch A.E., Koepsell H. (1997). Cloning and Characterization of Two Human Polyspecific Organic Cation Transporters. DNA Cell Biol..

[3] Gründemann D., Gorboulev V., Gambaryan S., Veyhl M., Koepsell H. (1994). Drug excretion mediated by a new prototype of polyspecific transporter. Nature.

[4] Green R.M., Lo K., Sterritt C., Beier D.R. (1999). Cloning and functional expression of a mouse liver organic cation transporter. Hepatology.

[5] Schmitt A., Mössner R., Gossmann A., Fischer I.G., Gorboulev V., Murphy D.L., Koepsell H., Lesch K.P. (2003). Organic cation transporter capable of transporting serotonin is up-regulated in serotonin transporter-deficient mice. J. Neurosci. Res..

[6] Wang D.-S., Jonker J.W., Kato Y., Kusuhara H., Schinkel A.H., Sugiyama Y. (2002). Involvement of Organic Cation Transporter 1 in Hepatic and Intestinal Distribution of Metformin. J. Pharmacol. Exp. Ther..

[7] Shu Y., Brown C., Castro R.A., Shi R.J., Lin E.T., Owen R.P., Sheardown S.A., Yue L., Burchard E.G., Brett C.M. (2008). Effect of genetic variation in the organic cation transporter 1, OCT1, on metformin pharmacokinetics. Clin. Pharmacol. Ther..

[8] Tzvetkov M.V., Vormfelde S.V., Balen D., Meineke I., Schmidt T., Sehrt D., Sabolić I., Koepsell H., Brockmöller J. (2009). The Effects of Genetic Polymorphisms in the Organic Cation Transporters OCT1, OCT2, and OCT3 on the Renal Clearance of Metformin. Clin. Pharmacol. Ther..

[9] Tzvetkov M.V., Matthaei J., Pojar S., Faltraco F., Vogler S., Prukop T., Seitz T., Brockmöller J. (2018). Increased Systemic Exposure and Stronger Cardiovascular and Metabolic Adverse Reactions to Fenoterol in Individuals with Heritable *OCT1* Deficiency. Clin. Pharmacol. Ther..

[10] Matthaei J., Kuron D., Faltraco F., Knoch T., Dos Santos Pereira J.N., Abu Abed M., Prukop T., Brockmöller J., Tzvetkov M.V. (2016). OCT1 mediates hepatic uptake of sumatriptan and loss-of-function *OCT1* polymorphisms affect sumatriptan pharmacokinetics. Clin. Pharmacol. Ther..

[11] Tzvetkov M.V., Saadatmand A.R., Lotsch J., Tegeder I., Stingl J.C., Brockmöller J. (2011). Genetically Polymorphic OCT1: Another Piece in the Puzzle of the Variable Pharmacokinetics and Pharmacodynamics of the Opioidergic Drug Tramadol. Clin. Pharmacol. Ther..

[12] Chen J., Brockmöller J., Seitz T., König J., Tzvetkov M.V., Chen X. (2017). Erratum to: Tropane alkaloids as substrates and inhibitors of human organic cation transporters of the SLC22 (OCT) and the SLC47 (MATE) families. Biol. Chem..

[13] Bourdet D.L., Pritchard J.B., Thakker D.R. (2005). Differential Substrate and Inhibitory Activities of Ranitidine and Famotidine toward Human Organic Cation Transporter 1 (hOCT1; SLC22A1), hOCT2 (SLC22A2), and hOCT3 (SLC22A3). J. Pharmacol. Exp. Ther..

[14] Meyer M.J., Seitz T., Brockmöller J., Tzvetkov M.V. (2017). Effects of genetic polymorphisms on the OCT1 and OCT2-mediated uptake of ranitidine. PLoS ONE.

[15] Hendrickx R., Johansson J.G., Lohmann C., Jenvert R.-M., Blomgren A., Börjesson L., Gustavsson L. (2013). Identification of Novel Substrates and Structure–Activity Relationship of Cellular Uptake Mediated by Human Organic Cation Transporters 1 and 2. J. Med. Chem..

[16] European Medicines Agency (2012). Guideline on the Investigation of Drug Interactions.

[17] U.S. Food and Drug Administration (2020). In Vitro Drug Interaction Studies—Cytochrome P450 Enzyme- and Transporter-Mediated Drug Interactions: Guidance for Industry.

[18] Zamek-Gliszczynski M.J., Taub M.E., Chothe P.P., Chu X., Giacomini K.M., Kim R.B., Ray A.S., Stocker S.L., Unadkat J.D., Wittwer M.B. (2018). Transporters in Drug Development: 2018 ITC Recommendations for Transporters of Emerging Clinical Importance. Clin. Pharmacol. Ther..

[19] Zamek-Gliszczynski M.J., Giacomini K.M., Zhang L. (2018). Emerging Clinical Importance of Hepatic Organic Cation Transporter 1 (OCT1) in Drug Pharmacokinetics, Dynamics, Pharmacogenetic Variability, and Drug Interactions. Clin. Pharmacol. Ther..

[20] European Medicines Agency (2009). ICH Guideline M3(R2) on Non-Clinical Safety Studies for the Conduct of Human Clinical Trials and Marketing Authorisation for Pharmaceuticals: Step 5.

[21] U.S. Food and Drug Administration (1938). Federal Food, Drug, and Cosmetic Act: FFDCA, FD&C Act. United States Code.

[22] Khanna C., Lindblad-Toh K., Vail D., London C., Bergman P., Barber L., Breen M., Kitchell B., McNeil E., Modiano J.F. (2006). The dog as a cancer model. Nat. Biotechnol..

[23] European Pet Food Industry Federation (2020). Facts & Figures 2020: European Overview.

[24] American Veterinary Medical Association (2018). AVMA Pet Ownership and Demographics Sourcebook: 2017–2018 Edition.

[25] Bleasby K., Castle J.C., Roberts C.J., Cheng C., Bailey W.J., Sina J.F., Kulkarni A.V., Hafey M.J., Evers R., Johnson J.M. (2006). Expression profiles of 50 xenobiotic transporter genes in humans and pre-clinical species: A resource for investigations into drug disposition. Xenobiotica.

[26] Gui C., Hagenbuch B. (2010). Cloning/characterization of the canine organic anion transporting polypeptide 1b4 (Oatp1b4) and classification of the canine OATP/SLCO members. Comp. Biochem. Physiol. Part C Toxicol. Pharmacol..

[27] Shu Y., Bello C.L., Mangravite L.M., Feng B., Giacomini K.M. (2001). Functional characteristics and steroid hormone-mediated regulation of an organic cation transporter in Madin-Darby canine kidney cells. J. Pharmacol. Exp. Ther..

[28] Meyer M.J., Tzvetkov M.V. (2021). OCT1 Polyspecificity—Friend or Foe?. Front. Pharmacol..

[29] Meyer M.J., Tuerkova A., Römer S., Wenzel C., Seitz T., Gaedcke J., Oswald S., Brockmöller J., Zdrazil B., Tzvetkov M.V. (2020). Differences in Metformin and Thiamine Uptake between Human and Mouse Organic Cation Transporter 1: Structural Determinants and Potential Consequences for Intrahepatic Concentrations. Drug Metab. Dispos..

[30] Meyer M.J., Schreier P.C.F., Basaran M., Vlasova S., Seitz T., Brockmöller J., Zdrazil B., Tzvetkov M.V. (2022). Amino acids in transmembrane helix 1 confer major functional differences between human and mouse orthologs of the polyspecific membrane transporter OCT1. J. Biol. Chem..

[31] Barrett T., Wilhite S.E., Ledoux P., Evangelista C., Kim I.F., Tomashevsky M., Marshall K.A., Phillippy K.H., Sherman P.M., Holko M. (2013). NCBI GEO: Archive for functional genomics data sets—Update. Nucleic Acids Res..

[32] Athar A., Füllgrabe A., George N., Iqbal H., Huerta L., Ali A., Snow C., Fonseca N., Petryszak R., Papatheodorou I. (2019). ArrayExpress update—From bulk to single-cell expression data. Nucleic Acids Res..

[33] Robinson J.T., Thorvaldsdóttir H., Winckler W., Guttman M., Lander E.S., Getz G., Mesirov J.P. (2011). Integrative genomics viewer. Nat. Biotechnol..

[34] Karakus E., Prinzinger C., Leiting S., Geyer J. (2021). Sequencing of the Canine Cytochrome P450 CYP2C41 Gene and Genotyping of Its Polymorphic Occurrence in 36 Dog Breeds. Front. Vet. Sci..

[35] Nies A.T., Koepsell H., Winter S., Burk O., Klein K., Kerb R., Zanger U.M., Keppler D., Schwab M., Schaeffeler E. (2009). Expression of organic cation transporters OCT1 (SLC22A1) and OCT3 (SLC22A3) is affected by genetic factors and cholestasis in human liver. Hepatology.

[36] Floerl S., Kuehne A., Hagos Y. (2020). Functional and Pharmacological Comparison of Human, Mouse, and Rat Organic Cation Transporter 1 toward Drug and Pesticide Interaction. Int. J. Mol. Sci..

[37] Dresser M.J., Gray A.T., Giacomini K.M. (2000). Kinetic and selectivity differences between rodent, rabbit, and human organic cation transporters (OCT1). J. Pharmacol. Exp. Ther..

[38] Morse B.L., Kolur A., Hudson L.R., Hogan A.T., Chen L.H., Brackman R.M., Sawada G.A., Fallon J.K., Smith P.C., Hillgren K.M. (2020). Pharmacokinetics of Organic Cation Transporter 1 (OCT1) Substrates in Oct1/2 Knockout Mice and Species Difference in Hepatic OCT1-Mediated Uptake. Drug Metab. Dispos..

[39] Meyer M.J., Neumann V.E., Friesacher H.R., Zdrazil B., Brockmöller J., Tzvetkov M.V. (2019). Opioids as Substrates and Inhibitors of the Genetically Highly Variable Organic Cation Transporter OCT1. J. Med. Chem..

[40] Mise M., Hashizume T., Matsumoto S., Terauchi Y., Fujii T. (2004). Identification of non-functional allelic variant of CYP1A2 in dogs. Pharmacogenetics.

[41] Mise M., Yadera S., Matsuda M., Hashizume T., Matsumoto S., Terauchi Y., Fujii T. (2004). Polymorphic expression of CYP1A2 leading to interindividual variability in metabolism of a novel benzodiazepine receptor partial inverse agonist in dogs. Drug Metab. Dispos..

[42] Tenmizu D., Endo Y., Noguchi K., Kamimura H. (2004). Identification of the novel canine CYP1A2 1117 C T SNP causing protein deletion. Xenobiotica.

[43] Geyer J., Janko C. (2012). Treatment of MDR1 mutant dogs with macrocyclic lactones. Curr. Pharm. Biotechnol..

[44] Mealey K.L., Bentjen S.A., Gay J.M., Cantor G.H. (2001). Ivermectin sensitivity in collies is associated with a deletion mutation of the mdr1 gene. Pharmacogenetics.

[45] Geyer J., Döring B., Meerkamp K., Ugele B., Bakhiya N., Fernandes C.F., Godoy J.R., Glatt H., Petzinger E. (2007). Cloning and Functional Characterization of Human Sodium-dependent Organic Anion Transporter (SLC10A6). J. Biol. Chem..

[46] Livak K.J., Schmittgen T.D. (2001). Analysis of relative gene expression data using real-time quantitative PCR and the 2(-Delta Delta C(T)) Method. Methods.

[47] Tzvetkov M.V., Saadatmand A.R., Bokelmann K., Meineke I., Kaiser R., Brockmoller J. (2012). Effects of OCT1 polymorphisms on the cellular uptake, plasma concentrations and efficacy of the 5-HT_3_ antagonists tropisetron and ondansetron. Pharm. J..

[48] Seitz T., Stalmann R., Dalila N., Chen J., Pojar S., Dos Santos Pereira J.N., Krätzner R., Brockmöller J., Tzvetkov M.V. (2015). Global genetic analyses reveal strong inter-ethnic variability in the loss of activity of the organic cation transporter OCT1. Genome Med..

[49] Smith P.K., Krohn R.I., Hermanson G.T., Mallia A.K., Gartner F.H., Provenzano M.D., Fujimoto E.K., Goeke N.M., Olson B.J., Klenk D.C. (1985). Measurement of protein using bicinchoninic acid. Anal. Biochem..

[50] Kim D., Paggi J.M., Park C., Bennett C., Salzberg S.L. (2019). Graph-based genome alignment and genotyping with HISAT2 and HISAT-genotype. Nat. Biotechnol..

[51] Sedlazeck F.J., Rescheneder P., Von Haeseler A. (2013). NextGenMap: Fast and accurate read mapping in highly polymorphic genomes. Bioinformatics.

[52] Lindblad-Toh K., Wade C.M., Mikkelsen T.S., Karlsson E.K., Jaffe D.B., Kamal M., Clamp M., Chang J.L., Kulbokas E.J., Zody M.C. (2005). Genome sequence, comparative analysis and haplotype structure of the domestic dog. Nature.

[53] Hoeppner M.P., Lundquist A., Pirun M., Meadows J.R.S., Zamani N., Johnson J., Sundström G., Cook A., Fitzgerald M.G., Swofford R. (2014). An Improved Canine Genome and a Comprehensive Catalogue of Coding Genes and Non-Coding Transcripts. PLoS ONE.

[54] Chen J., Swofford R., Johnson J., Cummings B.B., Rogel N., Lindblad-Toh K., Haerty W., Di Palma F., Regev A. (2019). A quantitative framework for characterizing the evolutionary history of mammalian gene expression. Genome Res..

[55] Li H. (2011). A statistical framework for SNP calling, mutation discovery, association mapping and population genetical parameter estimation from sequencing data. Bioinformatics.

