# Supplementary material for: Cloning and Functional Characterization of Dog OCT1 and OCT2: Another Step in Exploring Species Differences in Organic Cation Transporters
Source: Int J Mol Sci. 2022 May 4;23(9):5100. doi: 10.3390/ijms23095100 (PMC9102066; doi:10.3390/ijms23095100)
Supplement: Supplementary file 1 [file ijms-23-05100-s001.zip › ijms-1678927-supplementary.pdf]

Supplementary Material

Cloning and functional characterization of dog OCT1 and OCT2: another step in exploring species differences in organic cation transporters

Marleen J. Meyer, Simon Falk, Sarah Römer, Clarissa Prinzinger, Sabine Tacke, Joachim Geyer, Stefan Simm and Mladen V. Tzvetkov

Table of Contents

Supplementary Methods **2**

Supplementary Figures **3**

Figure S1 3

Figure S2 3

Figure S3 4

Figure S4 5

Figure S5 6

Figure S6 7

Figure S7 8

Supplementary Tables **9**

Table S1 9

Table S2 9

Table S3 9

Supplementary Methods

Generation of HEK293 cells stably overexpressing dog OCT1 and OCT2

HEK293 cells stably overexpressing dog OCT1 and OCT2 were generated by targeted chromosomal integration using the Flp-In™ system (Life Technologies) according to the manufacturer's instructions. To this end, 1×10^6 T-REx™-293 host cells were seeded in 6-well plates. One day later, the cells were transfected with 3.6 µg pOG44 helper plasmid (encoding the Flp recombinase) and 0.4 µg pcDNA5/FRT containing the gene of interest using 12 µL FuGene6 transfection reagent (Promega, Walldorf, Germany) in the absence of antibiotics according to the manufacturer's instructions. The next day, medium was changed to standard medium (DMEM/10% FBS/1% PS). The next day, cells were transferred into 10 mm Petri dishes and selection of transfected cells was initiated by adding the selection antibiotic hygromyicin B to a final concentration of 300 µg/ml at 24 h later. Medium was replaced with fresh standard medium containing 300 µg/ml hygromycin B 4 to 5 days later. Approximately 10 days after starting antibiotic selection, single monoclonal cell colonies were picked and transferred into 12-well plates and were subsequently expanded in 6-well plates and T25 tissue culture flasks (Sarstedt). When cells were passaged for the first time in T25 flasks, aliquots of approx. 1-2 × 10^6 cells were taken for DNA and RNA isolation. Cells were maintained in T25 flasks until they were validated and were then expanded into T75 flasks for long-term storage in liquid nitrogen and for use in transport experiments.

Characterization of HEK293 cells stably overexpressing dog OCT1 and OCT2

Successful genomic integration of the gene of interest after stable transfection was validated on DNA level using three integration-specific PCRs and primers listed in table S3 as follows. The hygromycin PCR was carried out using the Hot Start KOD Polymerase Kit (Sigma-Aldrich) according to the manufacturer's instructions in a final volume of 20 µL with 1x Q-solution (QIAGEN), 2.5 µM of each primer and 2 µL gDNA. Reaction conditions were 95°C initial denaturation for 2 min, followed by 35 cycles of 95°C for 30 sec, 64°C for 30 sec, and elongation at 72°C for 1 min, followed by a final elongation at 72°C for 10 min.

The gene of interest PCR was carried out using the Expand Long Template PCR System (Sigma-Aldrich) according to the manufacturer's instructions in a final volume of 28 µL with 1x Q-solution (QIAGEN), 1x buffer 1 and 3 µL gDNA. Reaction conditions were 94°C initial denaturation for 2 min, followed by 35 cycles of 96°C for 10 sec, 60°C for 20 sec, and elongation at 68°C for 5 min, followed by a final elongation at 68°C for 7 min.

The multiple integration PCR was carried out using the QIAGEN Multiplex PCR Kit according to the manufacturer's instructions in a final volume of 11 µL with 1x Q-solution (QIAGEN) and 1 µL gDNA. Reaction conditions were 95°C initial denaturation for 15 min, followed by 35 cycles of 94°C for 30 sec, 58°C for 90 sec, and elongation at 72°C for 2 min, followed by a final elongation at 72°C for 10 min. This PCR resulted in an amplicon only when the gene of interest was integrated multiple times, which was an exclusion criterion for cell validation.

Overexpression of the gene of interest was validated on RNA level using real-time qPCR as described in the methods section.

Supplementary Figures

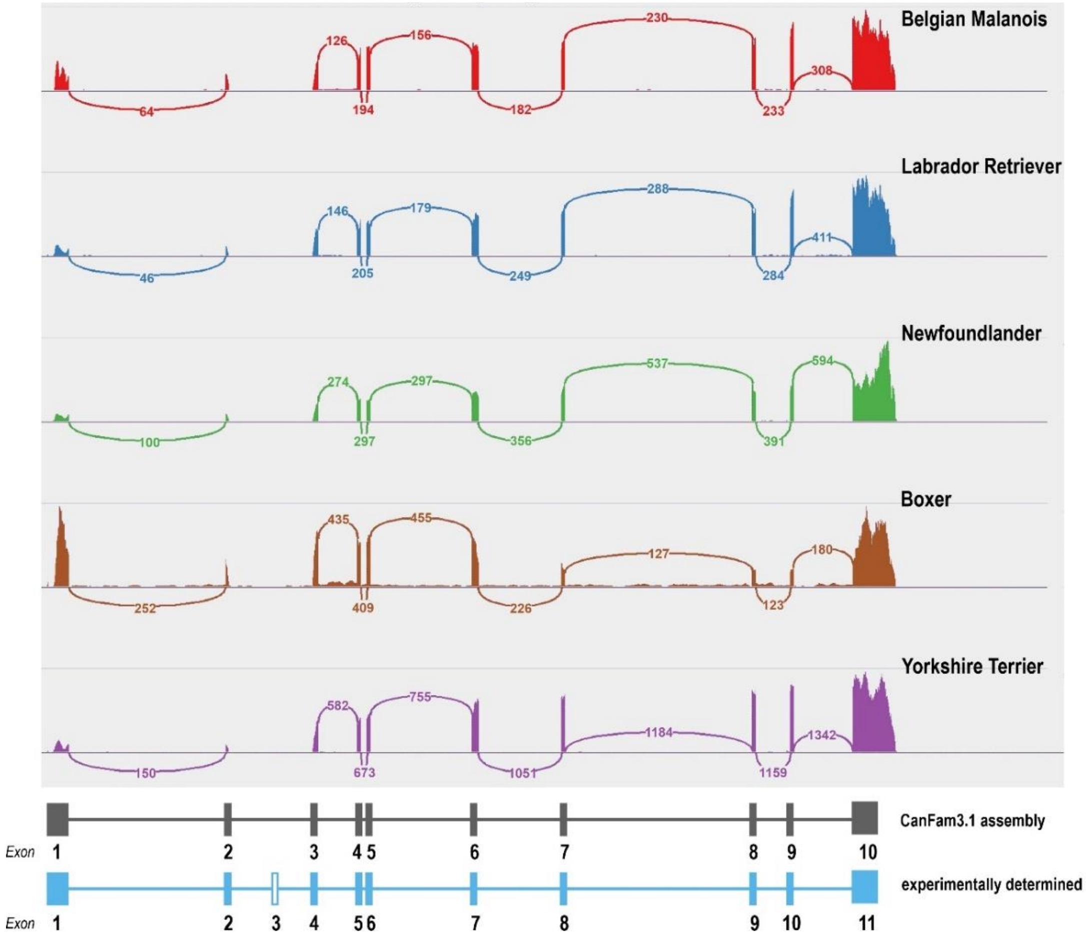

Figure S1. Gene structure of dog OCT1 in the liver of different dog breeds Sashimi plot showing OCT1 (SLC22A1) RNA-seq reads from liver of different dog breeds mapped onto chromosome 1 of CanFam3.1 dog genome assembly. Mapped exons are connected by lines, with the number indicating the number of splice junctions mapped. A splice junction is missing between exons 2 and 3.

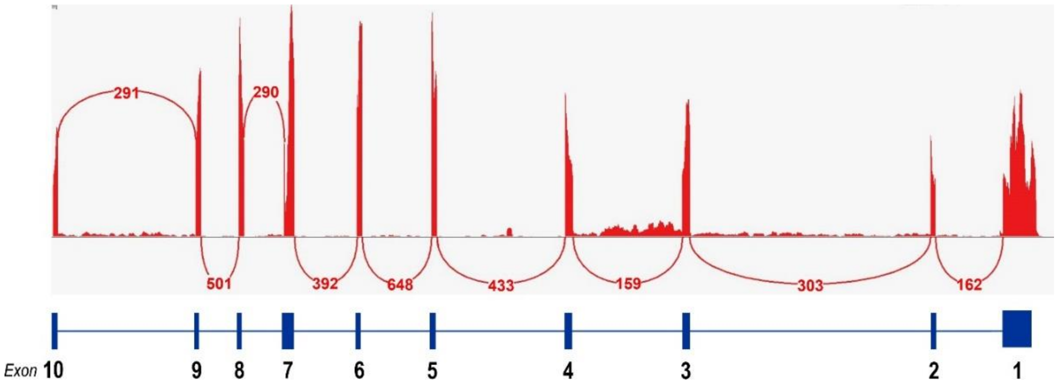

Figure S2. Gene structure of dog OCT2 Sashimi plot showing OCT2 (SLC22A2) RNA-seq reads from Beagle dog kidney mapped onto chromosome 1 of CanFam3.1 dog genome assembly. Mapped exons are connected by lines, with the number indicating the number of splice junctions mapped.

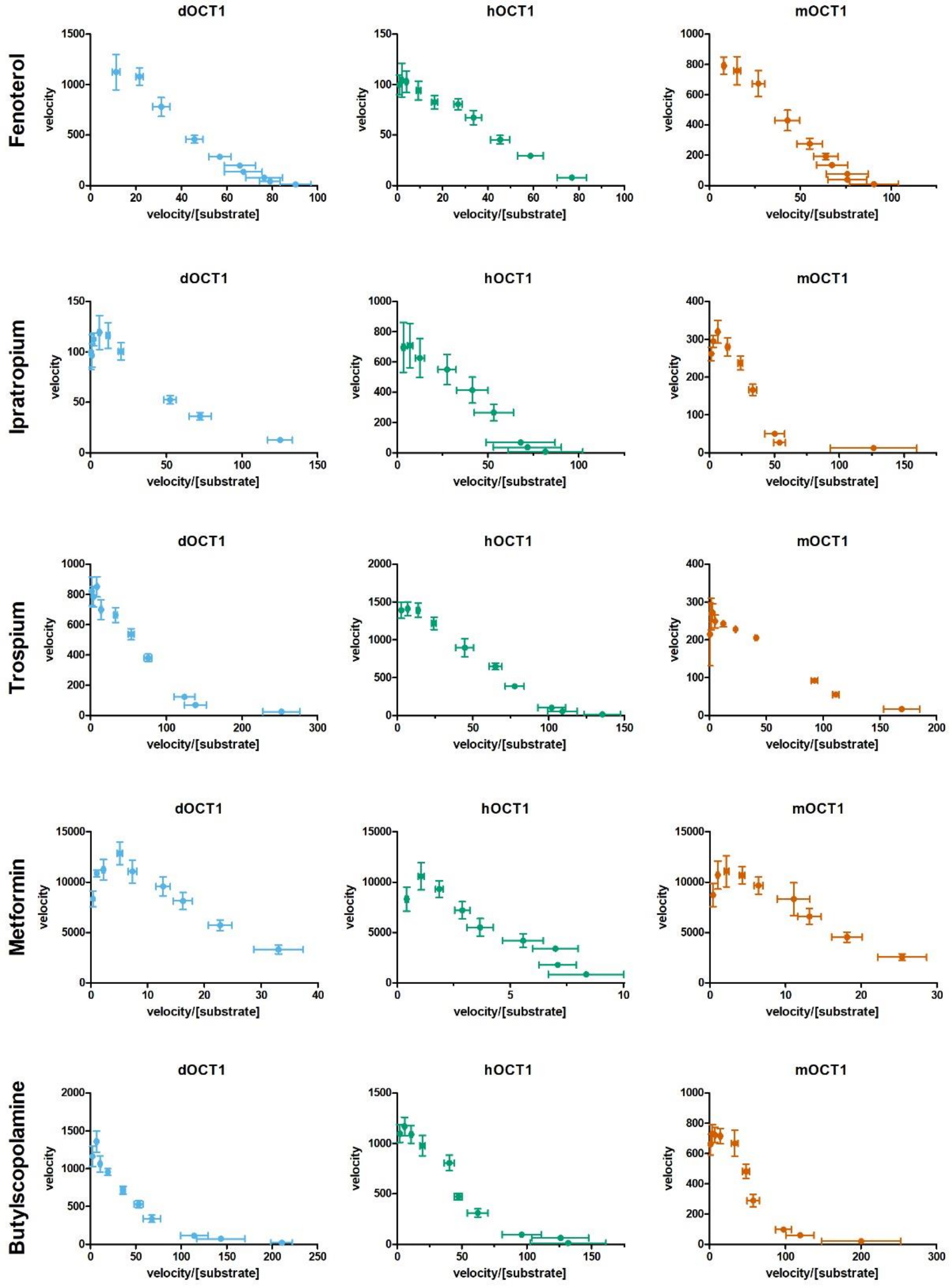

Figure S3. Eadie-Hofstee transformation of concentration-dependent substrate uptake by dog, human, and mouse OCT1 orthologs HEK293 cells stably transfected to overexpress dog (blue), human (green), and mouse (red) OCT1 were incubated for 2 min with increasing concentrations of fenoterol, ipratropium, trospium, metformin, and butylscopolamine. OCT1-mediated uptake was calculated by subtracting the uptake of control cells (pcDNA5) from the uptake of OCT1 overexpressing cells. Shown are means and standard errors of the means of at least three independent experiments. Please note the different ranges of x and y-axes. Michaelis-Menten representation of the dataset is shown in Figure 4.

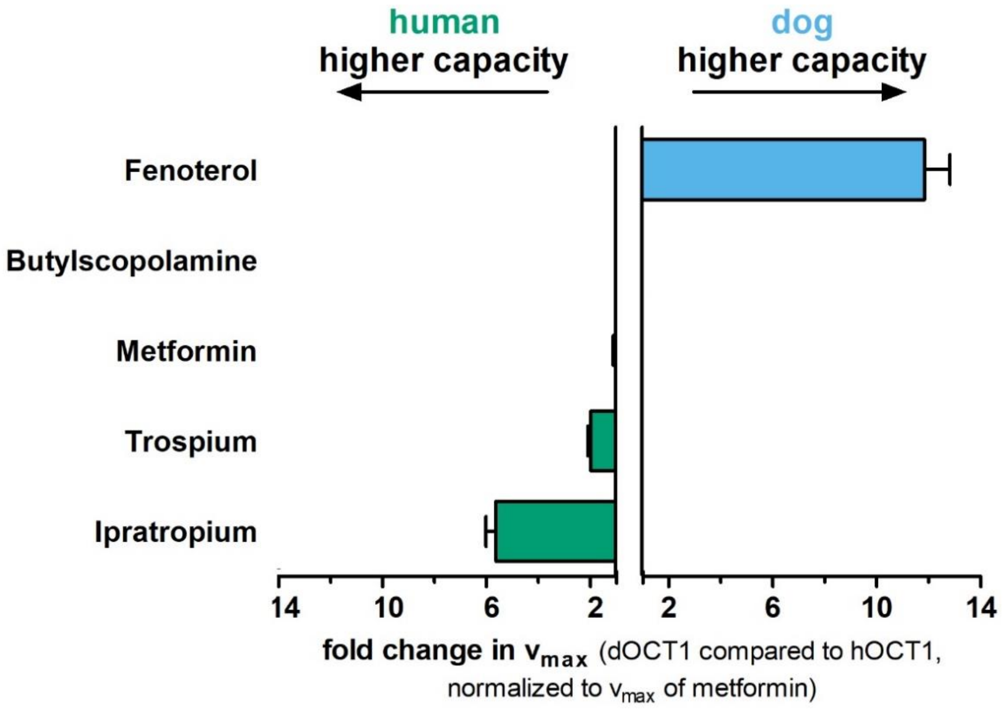

Figure S4. Comparison of transport capacity (v_{max}) between dog and human OCT1 orthologs Shown are the fold change in v_{max} between dog (blue) and human (green) OCT1 for the substrates shown in Figure 4. V_{max} values of individual experiments were normalized to the v_{max} for metformin. Shown are means and standard errors of the means of at least three independent experiments.

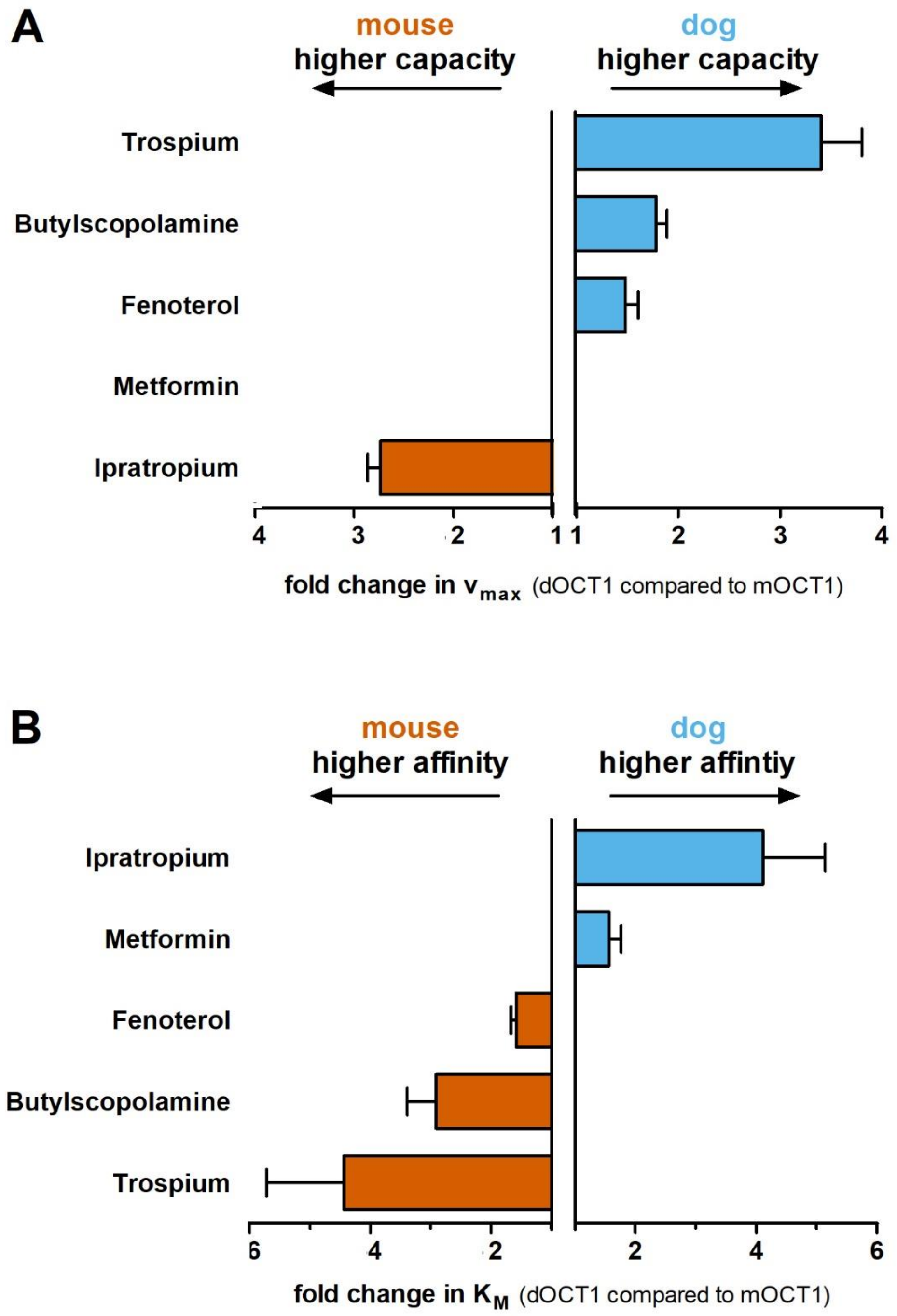

Figure S5. Comparison of transport capacity (v_{max}, A) and affinity (K_M, B) between dog and mouse OCT1 orthologs Shown are the fold change in v_{max} and K_M between dog (blue) and mouse (red) OCT1 for the substrates shown in Figure 4. hown are means and standard errors of the means of at least three independent experiments.

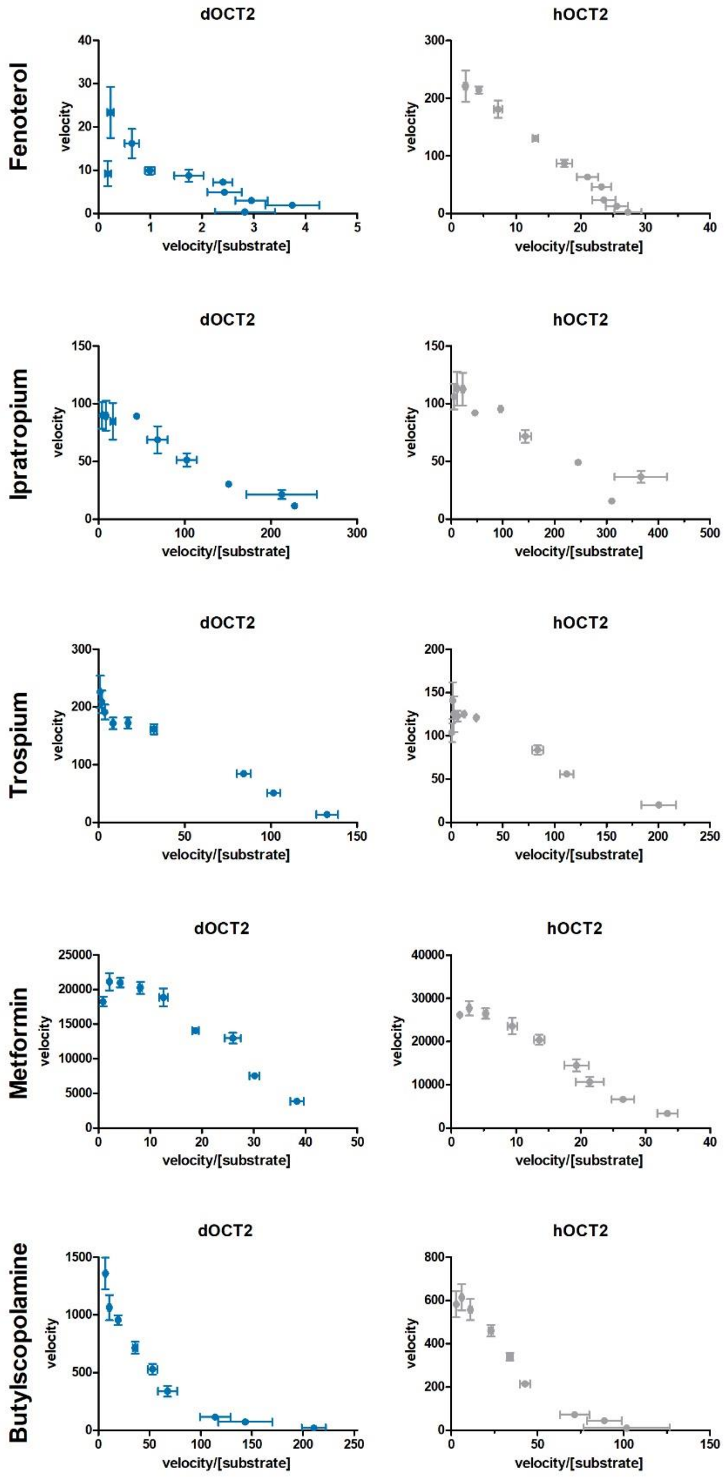

Figure S6. Eadie-Hofstee transformation of concentration-dependent substrate uptake by dog and human OCT2 orthologs HEK293 cells stably transfected to overexpress dog (blue) and human (grey) OCT2 were incubated for 2 min with increasing concentrations of fenoterol, ipratropium, trospium, metformin, and butylscopolamine. OCT1-mediated uptake was calculated by subtracting the uptake of control cells (pcDNA5) from the uptake of OCT2 overexpressing cells. Shown are means and standard errors of the means of at least three independent experiments. Please note the different ranges of x and y-axes. Michaelis-Menten representation of the dataset is shown in Figure 6.

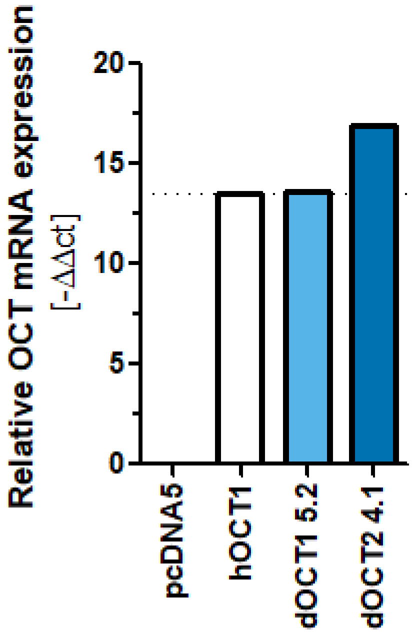

Figure S7. Relative mRNA expression of OCT1 or OCT2 for validation of stably transfected HEK293 cells Expression of OCT1 and OCT2 was measured after reverse transcription of mRNA extracted from stably transfected HEK293 cells using RT-qPCR. Expression was normalized to the expression of TBP. Cells stably transfected with hOCT1 and the empty vector pcDNA5 are positive and negative controls, respectively.

Supplementary Tables

Table S1. R^2 values of non-linear regression to Michaelis-Menten equation for transport measurements

Substrate	hOCT1	mOCT1	dOCT1	hOCT2	dOCT2
Fenoterol	0.8474	0.9271	0.9279	0.9533	0.3333
Ipratropium	0.9596	0.9280	0.8000	0.8580	0.7731
Trospium	0.9483	0.6796	0.9137	0.7656	0.8809
Metformin	0.7526	0.6271	0.6135	0.9494	0.9124
Butylscopolamine	0.9362	0.9023	0.9249	0.9384	0.4841

Table S2. Parameters of LC-MS/MS detection

Analyte	Quantifier precursor ion to product ion (m/z)	Retention time [min]	Internal standard (IS)	IS precursor ion to product ion (m/z)	IS retention time [min]	Mobile phase [% organic solvent]*	Flow [µL/min]	Injection volume [µL]
Fenoterol	304.1>107.1	3.14	Fenoterol-d6	310.3>109.1	3.11	12	450	10
Ipratropium	332.2>124.2	5.05	Atropine	290.2>124.4	4.54	15	300	10
Trospium	392.1>164.0	2.9	Trospium-d8	400.1>171.9	2.9	31.5	400	5
Metformin	130.1>71.0	1.66	Buformin	158.1>60.0	2.31	3	500	5
Butylscopolamine	360.3>138	4.76	Ipratropium	332.2>124.2	3.14	18	400	10

m/z, mass to charge ratio; * six parts acetonitrile + one part methanol

Table S3. Sequences of primers used for validation PCRs of stably transfected cells

PCR	Primer	Sequence (5′-3′)	Amplicon [bp]
Hygromycin	P_{SV40}	AGCTGTGGAATGTGTGTCAGTTAGG	519
	Hyg_r2	ACGCCCTCCTACATCGAAGCTGAAA	
Gene of interest	LacZ	CCTTCCTGTAGCCAGCTTTCATCAA	3041 (dOCT1)
	P_{CMV}	CCATGGTGATGCGGTTTTGGCAGTA	2978 (dOCT2)
Multiple integration	P_{FRT}_f	AATCGGGGGCTCCCTTTAGGGTTCC	214
	Hyg_r2	ACGCCCTCCTACATCGAAGCTGAAA	
